# Lipid Metabolism as a Source of Druggable Targets for Antiviral Discovery against Zika and Other Flaviviruses

**DOI:** 10.3390/ph12020097

**Published:** 2019-06-21

**Authors:** Miguel A. Martín-Acebes, Nereida Jiménez de Oya, Juan-Carlos Saiz

**Affiliations:** Department of Biotechnology, Instituto Nacional de Investigación y Tecnología Agraria y Alimentaria (INIA), 28040 Madrid, Spain; jdeoya@inia.es (N.J.d.O.); jcsaiz@inia.es (J.-C.S.)

**Keywords:** Zika virus, West Nile virus, dengue virus, flavivirus, antiviral, lipids

## Abstract

The Zika virus (ZIKV) is a mosquito-borne flavivirus that can lead to birth defects (microcephaly), ocular lesions and neurological disorders (Guillain-Barré syndrome). There is no licensed vaccine or antiviral treatment against ZIKV infection. The effort to understand the complex interactions of ZIKV with cellular networks contributes to the identification of novel host-directed antiviral (HDA) candidates. Among the cellular pathways involved in infection, lipid metabolism gains attention. In ZIKV-infected cells lipid metabolism attributed to intracellular membrane remodeling, virion morphogenesis, autophagy modulation, innate immunity and inflammation. The key roles played by the cellular structures associated with lipid metabolism, such as peroxisomes and lipid droplets, are starting to be deciphered. Consequently, there is a wide variety of lipid-related antiviral strategies that are currently under consideration, which include an inhibition of sterol regulatory element-binding proteins (SREBP), the activation of adenosine-monophosphate activated kinase (AMPK), an inhibition of acetyl-Coenzyme A carboxylase (ACC), interference with sphingolipid metabolism, blockage of intracellular cholesterol trafficking, or a treatment with cholesterol derivatives. Remarkably, most of the HDAs identified in these studies are also effective against flaviviruses other than ZIKV (West Nile virus and dengue virus), supporting their broad-spectrum effect. Considering that lipid metabolism is one of the main cellular pathways suitable for pharmacological intervention, the idea of repositioning drugs targeting lipid metabolism as antiviral candidates is gaining force.

## 1. Introduction

Zika virus (ZIKV) is a human pathogen transmitted by mosquitoes that caught major attention after its association with neonatal malformations (i.e., microcephaly) in Brazil in 2015 [1]. However, the consequences of ZIKV infection are not limited to pregnant women and newborns. The virus also induces a wide range of symptoms that go from fever, rash, headache, joint and muscle pain, and conjunctivitis to severe ocular lesions, and neurological disorders like Guillain-Barré syndrome [2,3]. The rapid expansion of ZIKV across the Americas led to the World Health Organization (WHO) to declare a Public Health Emergency of International Concern on the 1 February 2016. Since then, a great effort has been performed to develop effective control mechanisms. For instance, prophylactic (preventative) vaccines are being developed, and some of them have even undergone clinical trials [4]. Another front line of defense to combat this pathogen is the development of antiviral therapies [5]. There still is no specific antiviral therapy licensed for the treatment of ZIKV infection.

Antiviral development against ZIKV has to face specific challenges, such as safety for pregnant women, or the ability of the drugs to cross the blood-brain barrier (BBB) to combat the infection in nervous tissues [6]. Currently, there are two major lines of investigation for the development of antivirals against ZIKV: (i) The search for Direct Acting Antivirals (DAAs) that are based on interference with viral components (e.g., inhibitors of fusion, viral protease, or RNA dependent RNA polymerase); (ii) The identification of Host-Directed Antivirals (HDAs) that inhibit host factors co-opted for the virus to complete its infectious cycle. Theoretically, HDAs carry the advantage that they are less prone to the selection of resistant mutants, which could lead to the identification of broad-spectrum antivirals that are effective against taxonomically-related viruses [7,8,9]. During the last years, the knowledge on virus-host interactions has exponentially grown, revealing that the life cycle of many viruses, including ZIKV, is dependent upon host lipid metabolism [10,11]. This valuable information has unveiled potential antiviral targets that are now starting to be explored. It is expected that the detailed knowledge of the metabolic alterations (e.g., lipid metabolism) during ZIKV and other arboviral infections could help to design novel therapeutics, but also be useful to the identification of molecular biomarkers suitable for improved diagnostic [12]. This review summarizes the principal findings and the current status on the progress of the development of drugs that interfere with lipid metabolism and function as antivirals against ZIKV.

## 2. Biology of ZIKV and Its Connection with Cellular Lipids

**Figure 1:**
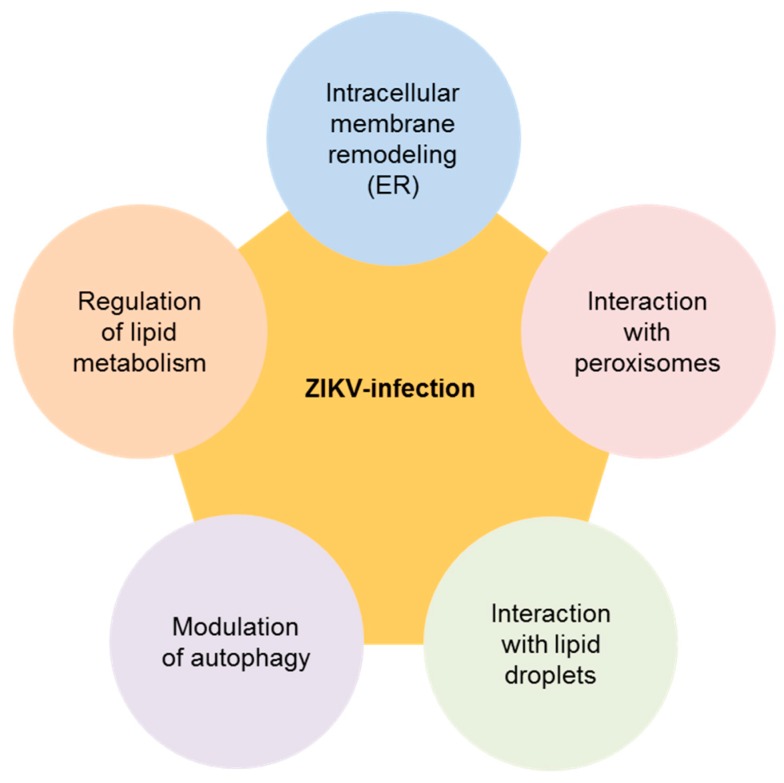
Multiple connections of the Zika virus (ZIKV) with lipid metabolism. The varied interactions of ZIKV with metabolic networks and lipid metabolism-related cellular structures are indicated. See the text for details.

ZIKV is a member of the genus *Flavivirus* within the family *Flaviviridae*. This genus includes 53 species of positive-sense single-stranded RNA viruses, including important pathogens, such as the dengue virus (DENV) and the West Nile virus (WNV) [13]. The genome (about 11 kb in length) encodes a single open reading frame translated into a viral polyprotein. This polyprotein undergoes cotranslational and posttranslational proteolytic cleavage by viral and host proteases to produce 10 mature proteins: Three structural proteins [C (capsid), M (membrane) and E (envelope)] and seven non-structural proteins (NS1, NS2A, NS2B, NS3, NS4A, NS4B and NS5) [3]. Notably, the majority of the structural and non-structural proteins of ZIKV exhibit transmembrane domains [3,14], and even NS1, which does not contain a transmembrane sequence, can associate with cellular membranes [15]. The interactions of viral proteins with lipids are important to complete different aspects of the ZIKV life-cycle, remarking the relevance of lipids during infection (Figure 1). As described for DENV and WNV [16,17,18,19], “omic” approaches have confirmed that ZIKV infection alters a significant number of lipid metabolites [20,21,22,23], suggesting that these metabolites are key players during the infection. Specifically, the analyses of the lipidomic and transcriptomic profiles of ZIKV-infected cells from different origins (microglia, mosquito or primary retinal pigment epithelium) reveal that ZIKV infection markedly modulates lipid metabolism. This remodeling of host lipid metabolism modulates the expression of sphingolipids like ceramide and sphingomyelin, glycerophospolipids (phosphoglycerides), such as phosphatidylcholine and phosphatidylserine, plasmenyl-phosphatidylethanolamines (also named plasmalogens), lysophospholipids such as lysophospha-tidylcholine, and (di)carboxylic acids, such as the undecanedioic and dodecanedioic acids [20,21,22,23]. Although in most cases the role in the infection of these lipids is still undefined, they would be presumably involved in key processes that go from virion biogenesis, membrane remodeling, genome replication, and neuronal differentiation, to the regulation of autophagy and apoptosis.

The involvement of lipids in flavivirus biology begins at the step of virion morphogenesis, because lipids constitute an essential component of virions. Mature ZIKV particles contain 17% lipid by weight (about 8000 lipid molecules in total), arranged in a lipid bilayer that constitutes the viral envelope [24]. Given that ZIKV does not encode the machinery necessary for lipid synthesis, these lipids are derived from host cell membranes. Specifically, the lipid envelope of ZIKV is hijacked from the membrane of the Endoplasmic Reticulum (ER), where genome replication and particle biogenesis are coupled. The analysis of the lipid envelope of the related flavivirus WNV indicates that specific lipid sorting is necessary for flavivirus morphogenesis, with virions enriched in sphingolipids (sphingomyelin) and reduced levels of phosphatidylcholine [17].

An important feature that ZIKV shares with other plus-strand RNA viruses is that replication is associated with a marked rearrangement of the cytoplasmic membranes. Actually, the ZIKV virus replicates in virus-induced membranous factories organized into specialized portions of the ER that undergo membrane invaginations containing pore-like openings toward the cytosol [25,26]. These structures are very similar to the vesicle packets (VPs) that are developed in the cells infected by DENV or WNV [27,28]. In this sense, the alteration of lipid metabolism can impair flavivirus genome replication and virion biogenesis, providing an interesting strategy for antiviral intervention (see Section 3). The membrane association of ZIKV non-structural proteins is important for viral replication and, in fact, NS2A, NS2B, NS4A, and NS4B are multi-pass transmembrane proteins. Accordingly, proper biogenesis and a membrane association of NS4A and NS4B is required for ZIKV replication [29]. In addition, NS2B, which is the activating co-factor of NS3 protease, must also anchor the viral protease to the ER [30]. As above mentioned, NS1 (a major host-interaction protein that functions in flaviviral replication, pathogenesis, and immune evasion) also associates to cellular membranes, despite the lack of a transmembrane domain [31].

Apart from lipids contained in the ER membranes, lipids in other cellular structures (peroxisomes and lipid droplets) have also been related to ZIKV infection. Several ZIKV proteins interact with proteins associated with peroxisomes and lipid transfer between ER and peroxisomes: ABCD3/PMP70; ACBD5, VAPA, VAPB, and PEX11BZIKV [32]. The viral NS2A is localized to peroxisomes, and its transient expression is sufficient to alter peroxisome morphology and distribution [32]. The analysis of ZIKV-infected patients also revealed increased levels of phosphatidylethanolamine and plasmalogens [23]. The synthesis of plasmalogens is dependent upon functional peroxisomes further supporting the role of these organelles during ZIKV-infection. The importance of peroxisomes in virus biology extend the results observed in other viral models such as influenza virus [33] or herpes viruses [34]. Considering that peroxisomes play important roles in both lipid metabolism and innate immunity, their connection with ZIKV infection could constitute an interesting target suitable for antiviral intervention. Another example of the dependence of ZIKV replication on cellular structures linked to lipid metabolism is its relation with lipid droplets. It has been documented that the capsid (C) protein of the ZIKV localized to lipid droplets [32,35] and that this association can be abolished by specific amino acid substitution in the C protein [36]. Although the consequences of this association have not been yet deciphered, it could be important for virion biogenesis as described for DENV [37].

At this point, it should also be mentioned that ZIKV-infection modulates cellular autophagy, presumably via the NS2B-NS3 cleavage of cellular factors and by the expression of NS4A and NS4B [38,39,40]. The interaction between ZIKV and the autophagic pathway seems to be complex, and data on the positive or negative effects of autophagy on the virus are controversial and probably dependent on cell types [41,42]. Most studies support a positive role for autophagy during infection, but there are also studies supporting the idea that the cholesterol derivative activators of autophagy can also reduce infection [43]. These discrepancies reinforce the need for a better understanding of the functional connection between lipids and autophagy during ZIKV infection. For the related DENV, the modulation of cellular autophagy in infected cells provides another connection with lipid/energy metabolism, membrane remodeling, and virus production [44,45]. Overall, these data suggest that flaviviruses like ZIKV depend on different aspects of lipid metabolism to complete their life cycle, which ideally could be exploited to identify suitable HDAs.

## 3. Lipids and Therapeutic Opportunities against ZIKV Infection

### 3.1. Lipid Metabolism Modulators

The dependence on lipid synthesis for flavivirus infection is extensively documented (revised in [10]) and specifically confirmed for ZIKV [20,21,22,32]. Taking into account these observations, it can be hypothesized that a downregulation of lipid synthesis using hypolipidemic agents could reduce ZIKV infection. Accordingly, several structurally-unrelated inhibitors of the sterol regulatory element-binding proteins (SREBP) pathway, a major regulator of lipid metabolism, reduces ZIKV-infection in cultured cells [46]. These compounds, namely nordihydroguaiaretic acid (NDGA), its methylated derivative tetra-O-methyl nordihydroguaiaretic acid (M_4_N), PF-429242, and fatostatin (Table 1) could constitute lead compounds for the development of antiviral therapies against ZIKV. Interestingly, SREBP inhibitors also inhibit the multiplication of the related WNV, DENV and Hepatitis C virus (another member of the family *Flaviviridae*), showing the potential broad-spectrum of antiviral therapies based on the inhibition of lipid metabolism [46,47,48,49]. Moreover, the antiviral potential of SREBP-dependent lipidomic reprogramming is also being explored for other taxonomically distant viruses, such as the Middle East respiratory syndrome coronavirus and the influenza A virus [50]. Another example of lipid metabolism modulators that exhibit anti-ZIKV activity is provided by the activators of the adenosine monophosphate-activated protein kinase (AMPK), one of the main cellular energy sensors that regulates glycolysis and lipid metabolism [51,52]. AMPK activators that show anti-ZIKV activity include specific small molecule compounds like PF-06409577, or indirect activators like metformin or 5-aminoimidazole-4-carboxamide ribonucleotide (AICAR) (Table 1) [51,52]. The activation of AMPK using these compounds also inhibits the infection of DENV and WNV [51,53], confirming the broad-spectrum antiviral potential of this kind of drugs. Results using a series of small-molecule inhibitors (PF-05175157, PF-05206574 and PF-06256254, Table 1) of acetyl-Coenzyme A carboxylase (ACC), the key enzyme of fatty acid metabolism, support the role of lipid metabolism and specifically fatty acid synthesis in ZIKV, DENV, and WNV infection [54]. One of these inhibitors, PF-05175157, which has undergone clinical trials in healthy volunteers (NCT01433380), and for the treatment of diabetes mellitus (NCT01792635), was tested against WNV in mouse models, showing a reduction of viremia and viral load in the kidney, suggesting the potential of these compounds for the treatment of flavivirus diseases. Interestingly, the activity of ACC is directly regulated by AMPK through phosphorylation. AMPK inhibits ACC enzymatic activity by phosphorylation at serine 79 (Ser79) [55]. Thus, the AMPK-ACC tandem could constitute a major druggable target for antiviral intervention against ZIKV and other related flaviviruses.

### 3.2. Interfering with Sphingolipid Metabolism

Sphingolipids constitute a lipid class that is defined by the presence of the long-chain amino alcohol sphingosine [64]. Sphingolipids, and specifically sphingomyelin, are particularly abundant in nervous tissues. These lipids play important roles in cellular physiology and disease [65], and have been associated with diverse steps of flavivirus infection both *in vitro* and *in vivo* using animal models [17,66,67,68]. In the case of ZIKV, a recent report has shown that infection in human fetal astrocytes can be reduced by a treatment with GW4869 (Table 1), a specific inhibitor of neutral sphingomyelinase-2, the enzyme that catalyzes the conversion from sphingomyelin to ceramide [56]. This study extends the previously reported antiflaviviral effect of this drug for the WNV and Usutu virus [17], highlighting again the potential broad-spectrum of HDAs targeting lipid metabolism.

### 3.3. Cholesterol and Derivatives

During flavivirus infection, cholesterol is involved in key steps, such as entry and membrane fusion, innate immunity, or virion biogenesis [69]. The importance of cholesterol for ZIKV infection can be extended to its mosquito vectors. For instance, the insect parasite bacteria Wolbachia modulates host-cell lipids [70] and disrupts cholesterol and vesicular trafficking, blocking the DENV and ZIKV life cycle [71,72,73]. Considering this dependence on cellular cholesterol for ZIKV infection, the statins, which are a class of inhibitors of cholesterol biosynthesis targeting the hydroxy-methyl-glutaryl CoA reductase (HMG-CoA reductase), a key enzyme of cholesterol biosynthesis, are proposed as potential antiviral candidates against ZIKV [74]. However, the potential of cholesterol as a pharmacological target against ZIKV is not restricted to the use of biosynthesis inhibitors. Recent reports point to cholesterol transport as a druggable target to combat ZIKV. The interference with cholesterol trafficking using imipramine (Table 1) inhibits the replication of ZIKV and other flaviviruses [57]. Later studies confirm this hypothesis, showing that benzamil (Table 1), an inhibitor of ABCG1, a membrane transporter of cholesterol, also reduces ZIKV-infectivity [22]. Thanks to their bioactive properties, cholesterol derivatives could also constitute therapeutic weapons to fight ZIKV.

Cholesterol-25-hydroxylase (CH25H) and its product 25-hydroxycholesterol (25-HC, Table 1) mediate protection against ZIKV infection and microcephaly in animal models [58]. The mechanism of action of 25-HC seems to be related to its immunostimulatory effect reducing inflammation and cell death caused by ZIKV infection and by directly blocking viral entry [58,59]. Thus, 25HC rises as an interesting candidate for a ZIKV therapeutic based on its safety and its ability to cross the BBB. Another cholesterol derivative that exhibits anti-ZIKV activity in neurons is 7-ketocholesterol (7-KC, Table 1) [43]. In this case, the proposed mechanism of inhibition of ZIKV infection is likely related to the induction of cellular autophagy by this compound.

### 3.4. Other Strategies

Daptomycin, a lipopeptide antibiotic that inserts into cell membranes rich in phosphatidylglycerol, is also described as a ZIKV inhibitor [60]. Finally, although this review is focused on therapeutic opportunities for the discovery of HDAs against ZIKV related to lipid metabolism, it should be noted that the lipids contained in the ZIKV-envelope, as a structural component of the virion, also provide suitable targets for the development of DAAs. For example, a treatment with the extract from *Aphloia theiformis*, an edible plant, can damage ZIKV virions and deform the viral shape, affecting viral entry [61]. Consistently, lipid envelope antiviral disruption appears as a complementary antiviral strategy to inhibit ZIKV. The small molecule CLR01 [62] or an amphipathic α-helical peptide also disrupt the virion envelope and reduce ZIKV-infectivity [63]. Importantly, the latter also exhibits a therapeutic effect on infected mice, showing the potential of these novel antiviral strategies.

## 4. Current Perspectives for Antiviral Therapies Related to Lipids

The above mentioned strategies to inhibit ZIKV infection provide the experimental proof-of-concept of the feasibility of strategies targeting lipids to combat viral infections. However, it is important to note that most of these studies are still in their initial stages and are mainly based on infections performed in cell culture. Remarkably, limited *in vivo* experiments also support the viability of this kind of approach for therapeutic intervention, as is the case of 25-HC or the peptide that disrupts the viral envelope [58,63]. Although it could seem risky to think in antiviral strategies based on lipid metabolism, because it constitutes one of the key cellular metabolic pathways, it is important to remember that lipid metabolism is actually a first order pharmaceutical target for the treatment of human disorders. Both SREBP and AMPK, antiviral targets already validated for ZIKV, currently constitute important pharmacological targets for human diseases (obesity, metabolic syndrome, type II diabetes and cancers). Some of these drugs have undergone diverse phases of clinical trials and others are even licensed for human use [75,76,77,78]. For instance, metformin, an indirect AMPK activator, is a drug licensed for humans and constitutes one of the most commonly prescribed drugs for the treatment of diabetes, supporting the safety of this kind of approach for the treatment of human illnesses [77,78]. Sphingomyelin metabolism modulators, and specifically the inhibitors of sphingomyelin to ceramide conversion, also constitute therapeutic targets currently under evaluation [79]. Likewise, drugs targeting cholesterol biosynthesis (i.e., statins) are commonly prescribed for the treatment of cardiovascular diseases and provide a further example of the safety and therapeutic success of drugs that interfere with lipid metabolism [80]. In this sense, imipramine, which inhibits ZIKV infection by altering cholesterol traffic, is an antidepressant also approved for human use, providing an additional example of a lipid modulator with an ability to cross the BBB that could be useful for the treatment of ZIKV [57]. Considering that most of the data supporting the antiviral potential of lipid-based antiviral strategies comes from cell cultures or small animal models, it could be interesting to test the reliability of these strategies in the real world. This could be initially addressed by taking advantage of lipid-lowering drugs already licensed (i.e., statins, metformin or imipramine). To this end, epidemiological surveys in patient cohorts from ZIKV-endemic regions could be performed. Both virological (viremia, viral load in urine, the severity of the infection) and biological parameters related to lipid metabolism such as the serum lipid profile, which is a biomarker for the severity of the infection of the related DENV [81], should be potentially included in these analyses.

Another important point that has to be taken into consideration is that most of the drugs targeting lipid metabolism here reviewed are effective against more than one flavivirus, confirming their broad-spectrum potential. Because most flaviviruses constitute neglected human pathogens, which complicates the development of specific antiviral strategies, repositioning drugs that target lipid metabolism as antiviral candidates could lead to more affordable broad-spectrum compounds. Although the utilization of antivirals to combat ZIKV infection, especially in pregnant women, raises multiple safety concerns, it should be noted that they could be useful for the treatment of other infected patients for the prevention of ZIKV-associated illnesses. Even more, effective antiviral treatments lowering the viral load should be useful to prevent ZIKV transmission by diminishing the risk of mosquito infection during blood-feeding, and by reducing the viral burden in the reproductive system of infected patients that could therefore also block any sexual transmission of the virus. It is important to remark that the antiviral effect exerted by some of the compounds targeting lipid metabolism in the infection models used is not very strong (showing a reduction of only about 1 log of virus production), so we still have to be cautious about the relevance of these drugs for more advanced studies. Nevertheless, lipid-targeting drugs could ideally be utilized, not only alone, but also in combinatorial therapies, together with other antivirals (i.e., DAAs), providing another weapon in the arsenal to combat the ZIKV.

In any case, and although a long way has yet to be completed before the implementation of these antiviral strategies in clinical practice, the current scenario and their potential benefits support future research efforts aimed to repositioning of lipid metabolism modulators as antiviral compounds.

## Figures and Tables

**Table 1:** Examples of some pharmacological inhibitors of ZIKV-infection related to lipid metabolism and function. (SREBP indicates sterol regulatory element-binding proteins, AMPK means adenosine-monophosphate activated kinase, ACC refers to acetyl-Coenzyme A carboxylase).

Drug Class or Proposed Target	Drug	Reference
SREBP pathway inhibitors	Nordihydroguaiaretic acid (NDGA)	[46]
	Tetra-*O*-methylnordihydro-guaiaretic acid(M_4_N)	[46]
	PF-429242	[46]
	Fatostatin	[46]
AMPK activators	PF-06409577	[51]
	Metformin	[52]
	AICAR	[52]
ACC inhibitors	PF-05175157	[54]
	PF-05206574	[54]
	PF-06256254	[54]
Neutral sphingomyelinase inhibitor	GSW4869	[56]
Intracellular cholesterol transport inhibitors	Benzamil	[22]
	Imipramine	[57]
Cholesterol derivatives	25-Hydroxycholesterol (25-HC)	[58,59]
	7-ketocholesterol (7-KC)	[43]
Lipopeptide antibiotic	Daptomycin	[60]
Lipid envelope disruptors	Extract from *Aphloia theiformis*	[61]
	CLR01	[62]
	Amphipathic α-helical peptide	[63]
